# Trends in Breast Cancer Incidence Rates by Age and Stage at Diagnosis in Gharbiah, Egypt, over 10 Years (1999–2008)

**DOI:** 10.1155/2013/916394

**Published:** 2013-10-24

**Authors:** Kelly A. Hirko, Amr S. Soliman, Ahmed Hablas, Ibrahim A. Seifeldin, Mohamed Ramadan, Mousumi Banerjee, Joe B. Harford, Robert M. Chamberlain, Sofia D. Merajver

**Affiliations:** ^1^Department of Epidemiology, University of Michigan School of Public Health, Ann Arbor, MI 48109, USA; ^2^Department of Epidemiology, College of Public Health, 984355 University of Nebraska Medical Center, Omaha, NE 68198, USA; ^3^Gharbiah Cancer Society, Tanta, Gharbiah, Egypt; ^4^Tanta Cancer Center, Tanta, Gharbiah, Egypt; ^5^Department of Biostatistics, University of Michigan School of Public Health, Ann Arbor, MI 48109, USA; ^6^Department of Health and Human Services, Center for Global Health, National Cancer Institute, National Institutes of Health, Bethesda, MD 20892, USA; ^7^Department of Epidemiology, University of Texas M.D. Anderson Cancer Center, Houston, TX 77030, USA; ^8^Department of Internal Medicine, University of Michigan Medical School, Ann Arbor, MI 48109, USA

## Abstract

*Background*. This study was undertaken to evaluate trends in breast cancer incidence in Egypt from 1999 to 2008 and to make projections for breast cancer occurrence for the years 2009–2015. *Patients and Methods*. We utilized joinpoint regression and average annual percent change (AAPC) measures with 95% confidence intervals (CI) to describe the trends in breast cancer incidence rates from the Gharbiah Cancer Registry by age and stage at diagnosis and to estimate expected breast cancer caseloads for 2009–2015. *Results*. From 1999 to 2008, the AAPC in breast cancer incidence rates in Gharbiah significantly increased among women 50 years and older and among localized tumors (AAPC %, 95% CI, 3.1% to 8.0%). Our results predict a significant increase in breast cancer caseloads from 2009 to 2015 among women aged 30–39 (AAPC %, 95% CI, 0.9% to 1.1%) and among women aged 40–49 years (AAPC %, 95% CI, 1.0% to 2.6%). *Conclusion*. These results have important implications for allocating limited resources, managing treatment needs, and exploring the consequences of prior interventions and/or changing risk factors in Egypt and other developing countries at the same stages of demographic and health transitions.

## 1. Introduction 

Breast cancer rates are increasing in developing countries, including Egypt, and are largely attributed to aging of the population, delay in time of first pregnancy, decrease in number of children and in breastfeeding, and a move toward high-calorie Western diets [1–4]. Although breast cancer incidence rates in Egypt are substantially lower than the rates in the United States and other developed countries [5–7], breast cancer is the most common cancer among women in Egypt [8]. Furthermore, the current demographic trends favor the likelihood that breast cancer will become an even greater public health concern in Egypt in the future.

Trends in the stage at diagnosis of breast cancer in the Gharbiah registry have not been reported, and this information is critical for evaluation of downstaging efforts. Detailed information on trends of breast cancer by stage of diagnosis may promote the reduction of disparities in the presentation of disease by focusing limited resources on the susceptible populations and can aid in our overall understanding of the etiology of breast cancer in a setting that differs in regard to its risk factor profile as compared to many developed countries. 

The specific aim of this study was to examine trends in breast cancer incidence by age, stage, and hormone receptor status in the Gharbiah registry from 1999 to 2008. Further, we evaluated the effect of possible changes in the population structure in order to make projections for breast cancer occurrence in Egypt for the years 2009–2015. 

## 2. Methods 

### 2.1. Gharbiah Population-Based Cancer Registry

The Gharbiah population-based cancer registry is located in Tanta, the capital city of the Gharbiah province. The population of Gharbiah is about 3.4 million and the registry was founded in 1998 as part of the Middle East Cancer Consortium (MECC) [1]. Data on cancer cases are actively collected from various sources throughout the province of Gharbiah. Breast cancer cases for this study came from hospitals, clinics, and pathology labs incorporating a comprehensive collection of all breast cancer cases in the Gharbiah region covered by this registry. Strict quality control checks are adhered to and data are entered using the International Agency for Research on Cancer (IARC) software CanReg4. Registrars are routinely trained in data extraction and entry methods and are periodically monitored by faculty of Emory School of Public Health, IARC, and MECC [1]. Coding of cancer is based on the International Classification of Diseases for Oncology 10th edition [9].

### 2.2. Study Population

A total of 7,049 cases of female breast cancer diagnoses were entered in the Gharbiah population-based cancer registry from 1999 to 2008. We excluded 52 cases with tumor behavior coded as uncertain or in situ, leaving 6,997 invasive cases for our study sample. For each case, the following information from routinely collected registry data was obtained for this analysis: age at diagnosis, estrogen receptor (ER) status, progesterone receptor (PR) status, summary stage at diagnosis, laterality of tumor, and basis for diagnosis. ER and PR status were determined by immunohistochemical results from the centers providing cases to the registry. We restricted our analysis on ER and PR status to the years 2001–2008, when this information was more routinely collected in the registry. The Surveillance, Epidemiology, and End Results (SEER) Summary Staging system was used to code stage at diagnosis [10]. Localized tumors were defined as those confined entirely to the organ of origin; regional tumors were those that extended into surrounding organs, tissues, or regional lymph nodes; and distant tumors were those that had spread to distant organs or lymph nodes. 

### 2.3. Statistical Analysis

Breast cancer incidence data from 1999 to 2008 were obtained from the Gharbiah Cancer registry. The average annual percent change (AAPC) in breast cancer rates was calculated using joinpoint regression for the age-specific incidence rates of breast cancer overall and by stage at diagnosis. The AAPC over the fixed interval of 1999–2008 is a weighted average of the slope coefficients of the underlying joinpoint regression line with the weights equal to the length of each segment over the interval [11]. 

Census data for female population in Gharbiah were obtained from the 1996 and 2006 Central Agency for Public Mobilization and Statistics (CAPMAS) census [12], and constant growth of the population was assumed to predict population estimates for the noncensal years using a linear regression model. The projected population numbers were multiplied by the most recent age-specific breast cancer incidence rates available from 2008 to estimate projected breast cancer caseloads by age group in Gharbiah, Egypt, from 2009 through 2015 accounting for population changes. Joinpoint regression models were fit to the predicted caseloads and AAPCs were utilized to describe trends in the projected future breast cancer cases. Data analysis was performed using Joinpoint Regression program [13] and SAS version 9.0 (SAS Institute Inc, Cary, NC); *P* ≤ 0.05 was used to determine statistical significance. The study was approved by the University of Michigan Institutional Review Board and the Gharbiah Cancer Center Ethics Committee.

## 3. Results

The majority of breast cancer cases during the study period were diagnosed among women aged 40–49 years (31.8%) and among women aged 50–59 years (29.8%) (Table 1). Most breast cancers were ER positive (36.9%) and PR positive (25.7%) (Table 1). Based on the limited hormonal receptor data, we found that the percentage of ER positive tumors decreased from 34.7% in 2001 to 27.2% in 2008 and the percentage of ER negative tumors increased from 11.0% in 2001 to 15.9% in 2008 (Table 1). The percentage of localized breast tumors increased over the study period, from 14.8% of tumors in 1999 to 21.4% of breast tumors in 2008 (Table 1).

### 3.1. Trends by Age at Diagnosis

**Figure 1:**
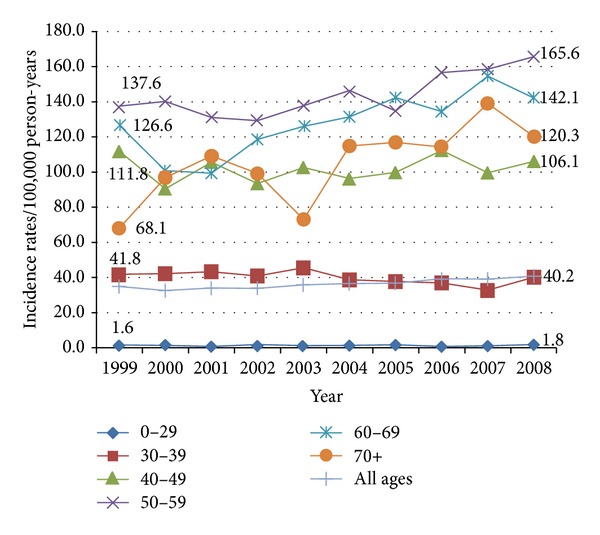
Breast cancer incidence rates/100,000 person-years by age group and year of diagnosis, 1999–2008.

Women aged 50–59 years had the highest overall breast cancer incidence rates through the years 1999–2008 (Figure 1). The overall breast cancer incidence rates increased in Gharbiah, Egypt, from 1999 to 2008 by an AAPC of 2.3% (95% CI = 1.5%, 3.0%) (Table 2). A significant increase in breast cancer incidence was evident among women aged 50 years and older, and the highest AAPC of 5.1% (95% CI = 1.2%, 9.2%) was noted among women aged 70 years and older (Table 2). We expect a significant increase in the breast cancer caseloads from 2009 to 2015 among women aged 30–39 years (AAPC = 1.0%, 95% CI 0.9%, 1.1%) and among women aged 40–49 years (AAPC = 1.8%, 95% CI = 1.0%, 2.6%) (Table 2).

### 3.2. Trends by Stage at Diagnosis

The AAPC in the overall breast cancer incidence rates increased for localized tumors by 5.5% (95% CI = 3.1%, 8.0%) and for regional tumors by 2.6% (95% CI = 1.0%, 4.3%), and there was a significant decrease in distant tumors among women aged 30–49 years (Table 3). The greatest significant increase in the incidence of localized tumors was evident among women aged 60–69 years with an AAPC of 9.4% (95% CI = 3.5%, 15.7%) (Table 3). The incidence of breast tumors diagnosed at a distant stage of disease decreased among women aged 30–39 years (AAPC = −11.3%, 95% CI = −19.6%, −2.1%) and among women aged 40–49 years (AAPC = −5.4%, 95% CI = −10.2%, −0.2%) (Table 3). The greatest expected increase in breast cancer caseloads are among women aged 50–59 years for localized (AAPC = 2.9%, 95% CI = 2.5%, 3.2%), regional (AAPC = 2.7%, 95% CI = 2.6%, 2.8%), and distant tumors (AAPC = 2.4%, 95% CI = 1.7%, 3.2%) (Table 3).

## 4. Discussion 

This study demonstrated a considerable increase in breast cancer incidence rates in Gharbiah, Egypt, from 1999 to 2008, particularly among women aged 50 years and older. While breast cancer incidence rates are increasing among older women, we found that the greatest expected increase in breast cancer caseloads from 2009 to 2015 is among women aged 30–49 years due to population changes. Further, our study noted a general decline in the incidence of distant tumors in Gharbiah, Egypt, from 1999 to 2008. 

Trends in reproductive factors and obesity associated with breast cancer favor the increase in breast cancer incidence in Egypt. For example, the fertility rate in Egypt is declining [14] and obesity is on the rise [14, 15]. Furthermore, in Egypt urban residence is clearly related to obesity risk [16–18] and the rate of urbanization from 2010 to 2015 is estimated at 2.1% annual rate of change [19]. Thus, the increasing urbanization of the population in Egypt could have implications on breast cancer trends through its effect on obesity. We found little information on physical activity trends in Egypt, although one report suggested that a large proportion of the population in Egypt is quite sedentary, particularly in urban areas [14]. Alcohol use is unlikely to account for the increase in breast cancer incidence in Egypt, where the majority of the population adheres to the Muslim religion, which prohibits use of alcohol. In summary, changes in the prevalence of established risk factors for breast cancer in Egypt may partially explain the increased incidence reported in this study, although future research should investigate other contributing factors.

The latent period between exposure to risk factors and the manifestation of disease may account to some extent for the observed trend of a statistically significant increase in breast cancer incidence only among women 50 years and older. For example, the effects of the Westernization of the Egyptian population may take several decades to develop into a detectable breast cancer increase. Therefore, the ill effects of the relatively recent adoption of a Western lifestyle may not have yet emerged in the younger age groups. Furthermore, there may be something inherent in the breast tissue of older women, which makes them more susceptible to the changing risk factor profile for breast cancer. These findings may also be attributed to the larger number of cases in the older women, providing greater power to demonstrate a statistically significant measure. 

Our finding of the greatest expected increase in breast cancer caseloads among younger women aged 30–49 largely reflects the increase in the population size among this age group; these results do not necessarily imply that screening efforts should target this age group. The incidence among younger age groups is very low and many women would have to be screened to find the cases. Therefore, in our opinion, awareness among younger women and education on breast self-exam may be the best approach to accomplish early detection among the younger age groups. 

Our finding of a general decline in incidence of distant tumors is encouraging given the emphasis on early detection and the screening efforts that have been occurring in Egypt over the study period. However, because of the overall population growth in Egypt, we can still expect a significant increase in breast tumors of all stages from 2009 to 2015. Therefore, while downstaging efforts are likely to be effective in reducing the incidence of breast tumors diagnosed at an advanced stage, Egypt must still be prepared to cope with the increased burden of diagnosing and treating breast tumors at all stages of disease.

There is evidence to suggest that hormonal subtypes of cancer differ in developing and developed countries, with ER positive tumors being more common in developed countries [20]. Hormonal receptor subtypes of breast cancer are important to consider due to their differential response to therapy, with better prognosis overall for ER positive tumors [21, 22]. Little information is available on recent trends of breast cancer by hormonal subtype in Egypt, though our previous study demonstrated higher incidence of ER positive tumors in urban areas as compared to rural areas in Egypt [23]. We were limited in our ability to evaluate trends in breast cancer by hormonal receptor status as part of this analysis due to missing data. However, our preliminary analysis suggested a significant increase in the incidence of ER negative tumors over this study period, with the greatest increase evident among women aged 50–59 years. Furthermore, we can expect an increase in ER negative tumor caseloads among women aged 70+. 

Most of the increase in breast cancer incidence in the United States has been due to an increase in ER positive breast cancer [24]. Reproductive factors that increase women's lifetime exposure to endogenous estrogens result in ER positive cancers, while smoking, radiation, and genetic risks are thought to give rise to ER negative cancers [25–27]. Alcohol consumption and family history of breast cancer has been shown to be associated with breast cancer regardless of ER status [28]. Thus, established risk factors for breast cancer associated with the Westernization of the population in Egypt would be more likely to explain an increase in ER positive tumors. However, this study suggests that a significant increase in the incidence of ER negative tumors is likely in Egypt, with the greatest expected increase in ER negative tumors from 2009 to 2015 among women 70 years and older. Future research should focus on risk factors that may illuminate the increasing trends of ER negative tumors in Egypt, especially among older women. This information is critical to cancer treatment planning and may also provide insight into the etiology of the hormonal subtypes of breast cancer. 

This study does have several important limitations that need to be considered. Most importantly, the stage at diagnosis and hormonal receptor status information was missing for a large proportion of the breast cancer cases in our analysis. The persistence of unknown stage and hormonal receptor status throughout the study years is disconcerting. Stage at diagnosis and hormonal receptor status information are critical metrics for treatment planning and for evaluation of cancer control programs. We believe that reporting of this information must be prioritized and that the specific challenges in reporting this information should be identified and ameliorated with urgency. Furthermore, we found statistically significant differences in the percentage of missing stage data across age groups and regions contributing cancer cases to the Gharbiah registry, with the greatest percentage of missing stage data coming from nonspecialized hospitals and clinics, pathology labs, and cases registered via death certificates only. Missing stage data was most notable among women aged 70+ (data not shown) and this may be due to the higher likelihood of diagnosis by fine needle aspirate (FNA) without tissue pathology available for staging among this age group. The incidence of breast cancer cases with unknown hormonal receptor status was previously shown to be similar from 1999 to 2006, and cases with unknown hormonal receptor status were similar to the overall breast cancer cases in the Gharbiah registry in regard to important baseline factors like age and stage at diagnosis [23]. However, we found that ER and PR information was more likely to be missing among women aged 70+ and among tumors diagnosed at a distant stage of disease (data not shown). Diagnosis by FNA among older women and those diagnosed at a distant stage of disease may explain this trend, as tissue would be unavailable for pathological staging or hormonal assays. 

The missing stage and hormonal status information could have limited our ability to demonstrate a significant measure of trend and could produce bias in our estimates of the trends in breast cancer occurrence in Egypt. The issue of missing hormonal receptor status is not unique to the Gharbiah registry. For example, one study of SEER data documented that between 1992 and 2007, 17% of cases had missing ER data and that the likelihood of missing data increased with increasing age at diagnosis and increasing stage of disease [29]. In summary, we must be extremely cautious in making inferences based on the observed trends in light of the fact that there was a significant amount of missing data for stage and hormonal receptor status that could have biased results.

A further limitation of this study is the fact that the breast cancer projections reported in this study assume stable screening practices, risk factor profiles, and constant incidence rates from 2008. Future predictions are affected by population growth and by aging and changing risk factors, which may be difficult to predict. Thus, while the projections reported in this study are based on statistical models, they should be interpreted with some caution. Moreover, the population figures for the years between the census were determined using linear interpolation, which assumes constant growth over these years. The accuracy of the calculated incidence rates would be affected if the actual population figures differ from our predicted values. Finally, registry-specific statistics are based on small numbers of cases per year observed in young women, with an inevitable high degree of variability. 

Strengths of this study include the use of a well-characterized and validated population-based registry data from a 10-year period. In addition, this study provides predictions for future trends, which are critical to cancer control and planning efforts in Egypt. Finally, this study provides important information on the progress of downstaging efforts in Egypt and also details trends in hormonal receptor status of tumors, which is critical for cancer treatment planning, especially in developing countries with limited treatment resources.

Breast cancer in Egypt is a growing public health concern and significant efforts should be directed to addressing the increasing burden of breast cancer in this part of the world. However, it is important to note that the breast cancer incidence rates we report for all age groups in Egypt are lower than what is reported for these age groups in the United States, including among younger women. Moreover, breast cancer rates in Egyptian women over 50 years are higher than the rates in Egyptian women under 50 years of age (Figure 1). Therefore, any impression that breast cancer is a disease of younger women in Egypt arises from the age distribution of the population. In this respect, Egypt is typical of many low- and middle-income countries. 

In conclusion, this study demonstrated that the breast cancer burden in Egypt will likely increase given the current population trends. The observed breast cancer incidence trends are generally consistent with the aging and Westernization of the population in Egypt. Our results have important implications for allocating limited resources, managing treatment needs, and exploring the consequences of prior interventions and/or changing risk factors in Egypt and other developing countries at the same stages of demographic and health transitions.

## Figures and Tables

**Table 1:** Characteristics of breast cancer cases (*n* = 6.997) by year of diagnosis in Gharbiah, Egypt, 1999–2008.

	Year of diagnosis										Overall *n* (%)	*P* value^a^
	1999 *n* (%)	2000 *n* (%)	2001 *n* (%)	2002 *n* (%)	2003 *n* (%)	2004 *n* (%)	2005 *n* (%)	2006 *n* (%)	2007 *n* (%)	2008 *n* (%)		
Age												
0–29	18 (2.9)	15 (2.5)	10 (1.6)	22 (3.5)	14 (2.0)	15 (2.1)	21 (2.9)	9 (1.2)	13 (1.7)	22 (2.6)	159 (2.3)	0.0051
30–39	103 (16.6)	107 (18.0)	110 (17.5)	104 (16.5)	115 (16.9)	103 (14.4)	100 (13.7)	101 (13.0)	88 (11.2)	113 (13.5)	1044 (14.9)	
40–49	215 (34.7)	180 (30.4)	214 (34.1)	195 (30.9)	223 (32.7)	216 (30.3)	229 (31.5)	263 (33.8)	235 (29.9)	253 (30.2)	2223 (31.8)	
50–59	164 (26.5)	177 (29.9)	172 (27.4)	178 (28.2)	198 (29.0)	218 (30.5)	209 (28.7)	244 (31.4)	255 (32.5)	271 (32.3)	2086 (29.8)	
60–69	94 (15.2)	76 (12.8)	77 (12.3)	91 (14.4)	101 (14.8)	109 (15.3)	117 (16.1)	110 (14.1)	129 (16.4)	122 (14.6)	1026 (14.7)	
70+	26 (4.2)	38 (6.4)	45 (7.2)	41 (6.5)	31 (4.6)	53 (7.4)	52 (7.1)	51 (6.6)	65 (8.3)	57 (6.8)	459 (6.6)	
Overall	620 (8.9)	593 (8.5)	628 (9.0)	631 (9.0)	682 (9.8)	714 (10.2)	728 (10.4)	778 (11.1)	785 (11.2)	838 (12.1)	6997 (100)	
ER^b^												
Positive	n/a	n/a	218 (34.7)	193 (30.6)	214 (31.4)	265 (37.1)	265 (36.4)	391 (50.3)	356 (45.4)	228 (27.2)	2130 (36.9)	<0.0001
Negative	n/a	n/a	69 (11.0)	92 (14.6)	112 (16.4)	112 (15.7)	127 (17.4)	148 (19.0)	137 (17.5)	133 (15.9)	930 (16.1)	
Missing	n/a	n/a	341 (54.3)	346 (54.8)	356 (52.2)	337 (47.2)	336 (46.2)	239 (30.7)	292 (37.2)	477 (56.9)	2724 (47.1)	
PR^c^												
Positive	n/a	n/a	126 (20.1)	110 (17.4)	135 (19.8)	157 (22.0)	194 (26.6)	314 (40.3)	272 (34.6)	181 (21.6)	1489 (25.7)	<0.0001
Negative	n/a	n/a	98 (15.6)	104 (16.5)	111 (16.3)	104 (14.6)	115 (15.8)	125 (16.1)	160 (20.4)	139 (16.6)	956 (16.5)	
Missing	n/a	n/a	404 (64.3)	417 (66.1)	436 (63.9)	453 (63.4)	419 (57.6)	339 (43.6)	353 (45.0)	518 (61.8)	3339 (57.7)	
Stage												
Localized	92 (14.8)	106 (17.9)	143 (22.8)	138 (21.9)	142 (20.8)	137 (19.2)	160 (22.0)	177 (22.8)	181 (23.1)	179 (21.4)	1455 (20.8)	<0.0001
Regional	314 (50.6)	275 (46.4)	313 (49.8)	323 (51.2)	327 (47.9)	360 (50.4)	359 (49.3)	387 (49.7)	365 (46.5)	453 (54.1)	3476 (49.7)	
Distant	87 (14.0)	95 (16.0)	83 (13.2)	71 (11.3)	100 (14.7)	94 (13.2)	77 (10.6)	77 (9.9)	95 (12.1)	55 (6.6)	834 (11.9)	
Missing	127 (20.5)	117 (19.7)	89 (14.2)	99 (15.7)	113 (16.6)	123 (17.2)	132 (18.1)	137 (17.6)	144 (18.3)	151 (18.0)	1232 (17.6)	
Laterality												
Right	267 (43.1)	255 (43.0)	261 (41.6)	269 (42.6)	287 (42.1)	327 (43.0)	307 (42.2)	351 (45.1)	319 (40.6)	405 (48.3)	3048 (43.6)	<0.0001
Left	284 (45.8)	273 (46.0)	324 (51.6)	332 (52.6)	344 (50.4)	340 (51.8)	370 (50.8)	373 (47.9)	398 (50.7)	399 (47.6)	3437 (49.1)	
Bilateral	5 (0.8)	3 (0.5)	6 (1.0)	5 (0.8)	9 (1.3)	5 (1.1)	8 (1.1)	5 (0.6)	2 (0.3)	5 (0.6)	53 (0.8)	
Missing	64 (10.3)	62 (10.5)	37 (5.9)	25 (4.0)	42 (6.2)	42 (6.0)	43 (5.9)	49 (6.3)	66 (8.4)	29 (3.5)	459 (6.6)	
Basis												
Histology	398 (64.2)	469 (79.1)	468 (74.5)	518 (82.1)	553 (81.1)	571 (80.0)	592 (81.3)	652 (83.8)	626 (79.8)	673 (80.3)	5520 (78.9)	<0.0001
FNAC^d^	168 (27.1)	83 (14.0)	142 (22.6)	99 (15.7)	105 (15.4)	118 (16.5)	113 (15.5)	107 (13.8)	138 (17.6)	150 (17.9)	1223 (17.5)	
Others	54 (8.7)	40 (6.7)	18 (2.9)	14 (2.2)	23 (3.4)	24 (3.4)	23 (3.2)	19 (2.4)	21 (2.7)	15 (1.8)	251 (3.6)	
Missing	0 (0.0)	1 (0.2)	0 (0.0)	0 (0.0)	1 (0.1)	1 (0.1)	0 (0.0)	0 (0.0)	0 (0.0)	0 (0.0)	3 (0.0)	

^a^ *P* value based on chi-square test.

^b^Estrogen receptor status.

^c^Progesterone receptor status.

^d^Fine needle aspiration cytology.

**Table 2:** Average annual percent change (AAPC) in breast cancer incidence rates/100,000 person-years by age group and year of diagnosis, 1999–2008, and predictions for breast cancer caseloads, years 2009–2015.

Age group	Years	AAPC^a^	LCL^b^	UCL^c^
0–29	1999–2008	0.0%	−6.9%	7.5%
30–39	1999–2008	−1.9%	−3.7%	0.0%
40–49	1999–2008	0.3%	−1.6%	2.2%
50–59	1999–2008	**2.3%**	**0.8%**	**3.7%**
60–69	1999–2008	**3.6%**	**1.2%**	**6.0%**
70+	1999–2008	**5.1%**	**1.2%**	**9.2%**
Overall	1999–2008	**2.3%**	**1.5%**	**3.0%**

0–29	2009–2015	−0.2%	−4.7%	4.6%
30–39	2009–2015	**1.0%**	**0.9%**	**1.1%**
40–49	2009–2015	**1.8%**	**1.0%**	**2.6%**
50–59	2009–2015	1.8%	−0.7%	4.5%
60–69	2009–2015	0.5%	−1.5%	2.5%
70+	2009–2015	1.1%	−1.2%	3.4%
Overall	2009–2015	1.4%	−0.2%	3.1%

^a^Average annual percent change.

^b^Lower confidence limit (95% confidence interval).

^c^Upper confidence limit (95% confidence interval).

Results in bold are statistically significant at the alpha 0.05 level.

**Table 3:** Average annual percent change (AAPC) in breast cancer incidence rates/100,000 person-years by summary stage and age group and predictions for breast cancer caseloads, years 2009–2015.

Stage	Age group	Years	AAPC^a^	LCL^b^	UCL^c^
Localized	0–29	1999–2008	−2.7%	−16.2%	13%
	30–39	1999–2008	−1.3%	−6.4%	4.1%
	40–49	1999–2008	**5.8%**	**2.4%**	**9.3%**
	50–59	1999–2008	3.1%	−1.8%	8.3%
	60–69	1999–2008	**9.4%**	**3.5%**	**15.7%**
	70+	1999–2008	18.2%	−1.7%	42.0%
	Overall	1999–2008	**5.5%**	**3.1%**	**8.0%**
Regional	0–29	1999–2008	−0.5%	−11.1%	11.4%
	30–39	1999–2008	−0.9%	−3.9%	2.3%
	40–49	1999–2008	−0.7%	−3.4%	2.0%
	50–59	1999–2008	**3.8%**	**1.2%**	**6.4%**
	60–69	1999–2008	**4.7%**	**1.8%**	**7.7%**
	70+	1999–2008	6.6%	−2.5%	16.6%
	Overall	1999–2008	**2.6%**	**1.0%**	**4.3%**
Distant	0–29	1999–2008	−45.7%	−76.2%	23.8%
	30–39	1999–2008	**−11.3%**	**−19.6%**	**−2.1%**
	40–49	1999–2008	**−5.4%**	**−10.2%**	**−0.2%**
	50–59	1999–2008	−2.2%	−9.4%	5.5%
	60–69	1999–2008	−4.9%	−11.4%	2.1%
	70+	1999–2008	3.8%	−8.6%	18.0%
	Overall	1999–2008	−4.0%	−8.2%	0.4%

Localized	0–29	2009–2015	0.0%	0.0%	0.0%
	30–39	2009–2015	0.9%	0.1%	1.7%
	40–49	2009–2015	2.1%	1.9%	2.4%
	50–59	2009–2015	2.9%	2.5%	3.2%
	60–69	2009–2015	1.0%	0.6%	1.4%
	70+	2009–2015	2.2%	0.9%	3.5%
	Overall	2009–2015	1.3%	1.2%	1.4%
Regional	0–29	2009–2015	1.5%	0.5%	2.5%
	30–39	2009–2015	1.0%	0.8%	1.2%
	40–49	2009–2015	2.0%	1.9%	2.2%
	50–59	2009–2015	2.7%	2.6%	2.8%
	60–69	2009–2015	1.1%	0.9%	1.3%
	70+	2009–2015	1.6%	1.0%	2.3%
	Overall	2009–2015	1.3%	1.3%	1.4%
Distant	0–29	2009–2015	0.0%	0.0%	0.0%
	30–39	2009–2015	0.0%	0.0%	0.0%
	40–49	2009–2015	2.1%	1.3%	3.0%
	50–59	2009–2015	2.4%	1.7%	3.2%
	60–69	2009–2015	2.3%	0.8%	3.8%
	70+	2009–2015	2.3%	0.2%	4.5%
	Overall	2009–2015	1.4%	1.2%	1.7%

^a^Average annual percent change.

^b^Lower confidence limit (95% confidence interval).

^c^Upper confidence limit (95% confidence interval).

Results in bold are statistically significant at the alpha 0.05 level.
